# Association of Alpha-Crystallin with Fiber Cell Plasma Membrane of the Eye Lens Accompanied by Light Scattering and Cataract Formation

**DOI:** 10.3390/membranes11060447

**Published:** 2021-06-15

**Authors:** Raju Timsina, Laxman Mainali

**Affiliations:** 1 Department of Physics, Boise State University, Boise, ID 83725, USA; rajutimsina@boisestate.edu; 2 Biomolecular Sciences Graduate Program, Boise State University, Boise, ID 83725, USA

**Keywords:** α-crystallin, fiber cell plasma membrane, lipids, cholesterol, cholesterol bilayer domains, integral membrane protein, lipid peroxidation, mutations, post-translational modifications, cataract

## Abstract

α-crystallin is a major protein found in the mammalian eye lens that works as a molecular chaperone by preventing the aggregation of proteins and providing tolerance to stress in the eye lens. These functions of α-crystallin are significant for maintaining lens transparency. However, with age and cataract formation, the concentration of α-crystallin in the eye lens cytoplasm decreases with a corresponding increase in the membrane-bound α-crystallin, accompanied by increased light scattering. The purpose of this review is to summarize previous and recent findings of the role of the: (1) lens membrane components, i.e., the major phospholipids (PLs) and sphingolipids, cholesterol (Chol), cholesterol bilayer domains (CBDs), and the integral membrane proteins aquaporin-0 (AQP0; formally MIP26) and connexins, and (2) α-crystallin mutations and post-translational modifications (PTMs) in the association of α-crystallin to the eye lens’s fiber cell plasma membrane, providing thorough insights into a molecular basis of such an association. Furthermore, this review highlights the current knowledge and need for further studies to understand the fundamental molecular processes involved in the association of α-crystallin to the lens membrane, potentially leading to new avenues for preventing cataract formation and progression.

## 1. Introduction

The eye lens’s main function is to focus light onto the retina in the back of the eye. The eye lens is avascular, avoiding light scattering, and is in a hypoxic environment, containing a lower level of oxygen than in any other organ in the human body (pO_2_ of 11 mm Hg) [1,2]. Light traveling through the human lens passes through approximately 2800 fiber cell plasma membranes [2]. The lens’s fiber cell plasma membrane consists of three major components: phospholipids (PLs) and sphingolipids, cholesterol (Chol), and the two major integral membrane proteins [3,4,5] such as aquaporin-0 (AQP0), also known as the major intrinsic protein 26 (MIP26, where 26 represents its location in the gel at 26 kDa) [6,7,8], and connexins (Cx43, Cx46, and Cx50) [9]. Moreover, the eye lens consists of large amounts of crystallin proteins [10,11,12].

**Figure 1:**
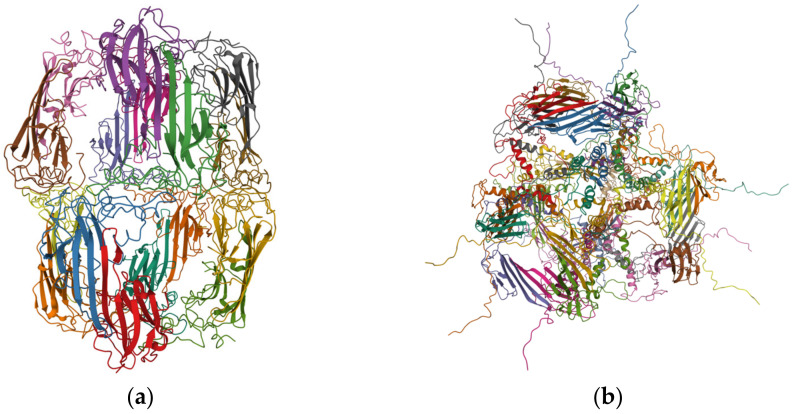
(**a**) Pseudo-atomic model of recombinant human αA-crystallin (reduced) 16-mer assembly. Image from the RCSB PDB (rcsb.org, accessed on: 8 April 2021) of PDB ID 6T1R [20] created with Mol* [28]. (**b**) Pseudo-atomic model of recombinant human αB-crystallin 24-mer assembly. Image from the RCSB PDB (rcsb.org, accessed on: 8 April 2021) of PDB ID 3J07 [21] created with Mol* [28].

Crystallins, i.e., α-, β-, and γ-crystallin, are water-soluble proteins found in a large concentration in the vertebrate eye lens [10,11,12]. Crystallins account for more than 90% of the lens proteins [10,11]. Unlike other proteins in the cell, crystallins, once synthesized, remain in the lens for the individual’s lifetime [13]. The primary function of crystallins is contributing to lens transparency and refractive properties [14]. α-crystallin accounts for up to 40% of the lens proteins [10,12]. In the human lens, α-crystallin remains in the oligomeric form comprising two types of subunits, αA- and αB-crystallin, in a roughly 3:1 molar ratio [15]. It is believed that the molar ratio of 3:1 of αA- and αB-crystallin increases α-crystallin’s stability [16]. αA-crystallin is found only in the lens, whereas αB-crystallin is found in the lens and many other tissues, including the heart, nervous system, striated muscles, and the kidney [17]. αA- and αB-crystallin consist of 173 and 175 amino acid residues, with ~60% sequence similarity [14], and have a mass of 19.9 and 20.2 kDa, respectively [18]. The oligomeric form of α-crystallin has a mass range from 300 to 900 kDa [19]. The use of combined techniques, such as electron microscopy, size exclusion chromatography, single-particle 3D reconstruction, analytical ultracentrifugation, and dynamic light scattering, determined the structure of the native α-crystallin to be an asymmetrical, bean-like shape, with a dense core and filamentous [19]. Recently, detailed molecular-level structures of the recombinant human αA-crystallin (reduced) 12-, 16-, and 20-meric assemblies were determined by combining cryo-electron microscopy, nuclear magnetic resonance (NMR) spectroscopy, cross-linking/mass spectrometry, and molecular modeling [20]. Similarly, a detailed molecular-level structure of the recombinant human αB-crystallin 24-mer structure was determined by combining solid-state NMR, small-angle X-ray scattering, and electron microscopy [21]. Figure 1a,b show the molecular-level structure of recombinant human αA-crystallin (reduced) 16-mer [20] and αB-crystallin 24-mer [21], respectively. The concentration of α-crystallin in the center of the human lens reaches up to 450 mg/mL [22]. α-crystallin functions as a molecular chaperone [23,24], increasing the tolerance to stress and preventing the precipitation of denatured proteins [25], whereas β- and γ- crystallins maintain the structure and refractive properties of the lens [26]. These functions of crystallins are significant for maintaining lens transparency and preventing cataract formation [27].

The causes of cataract, an opaqueness in the eye lens causing blurry vision, are aging [29,30], eye injury [31,32], genetics [33,34], radiation [35,36,37], high myopia [38,39,40], smoking [41], medications (e.g., statin and corticosteroid medicine to reduce Chol) [42,43,44,45,46,47,48,49], significant alcohol consumption [50,51,52,53], obesity [54,55], hypertension [56], and diabetes [57,58]. Among these causes, aging is the most common cause of cataract formation in which the association of α-crystallin to the eye lens’s fiber cell plasma membrane increases progressively [59,60,61,62,63,64,65]. With age and at the onset of cataract formation, water-soluble α-crystallin slowly depletes to the insoluble aggregates [61,63,64,66,67,68,69]; however, the nature of such insoluble aggregates is not well characterized [63,67,68,69,70,71,72,73,74]. In the human lens nucleus, the most pronounced conversion of soluble to insoluble α-crystallin and the corresponding conversion of soluble to insoluble high molecular weight (HMW) protein occurs after the age of 40 [63,67,68]. α-crystallin’s aggregation is an important factor in cataract formation [18,75]. In aged human lenses, all water-insoluble crystallins are membrane-bound [61]. With age, α-crystallin is associated with other lens proteins, forming higher molecular weight complexes (HMWCs) [76,77,78]. HMWCs further associate with the fiber cell plasma membrane [66], accompanied by light scattering and cataract formation [59,60,79]. A longitudinal clinical study [62] performed with 45 patients (66 eyes) aged 34–79 years using dynamic light scattering (DLS) shows that the higher levels of membrane-bound α-crystallin, with a corresponding decrease in the unbound α-crystallin concentration in the lens cytoplasm, are associated with nuclear cataract formation and progression. A hypothesis is that membrane-bound α-crystallin contributes to the formation of nuclear cataracts by obstructing the membrane pores and forming a barrier to diffusion [30,63,64]. Zhao et al. [75] showed that lanosterol, an amphipathic molecule enriched in the lens, reverses lens protein aggregation and maintains lens transparency in in-vivo dogs and dissected rabbits’ cataractous lenses in-vitro. Based upon the in-vitro, in-vivo, and ex-vivo studies on the mouse, Makley et al. [80] reported a class of molecules called pharmacological chaperones (PCs), namely compound 29, that bind a specific region of αA and αB, restoring solubility of these proteins and partially reversing their aggregation and cataract formation. Combining results of the clinical study on humans [62] and in-vitro, in-vivo, and ex-vivo studies on animals [75,80], we suggest that α-crystallin aggregates likely associate with lens membranes accompanied by light scattering and cataract formation.

The precise mechanism of how α-crystallin associates with the lens membrane causing cataract formation is still under investigation. This review summarizes the current and previous findings of the role of the lens membrane components, such as major PLs and sphingolipids, Chol, cholesterol bilayer domains (CBDs), and the integral membrane proteins (AQP0, also known as Major Intrinsic Protein 26 (MIP26), and connexins), on the association of α-crystallin to the lens membrane causing cataract formation. Moreover, we discuss the lipid peroxidation and α-crystallin mutations and post-translational modifications (PTMs) that occur with age and cataract formation. This review summarizes the current knowledge and future studies needed for a molecular-level understanding of the association of α-crystallin with the fiber cell plasma membrane of the eye lens causing cataract formation and progression.

## 2. Binding of α-Crystallin to Lipids (Phospholipids (PLs) and Sphingolipids) of the Eye Lens Fiber Cell Plasma Membrane

Lipid composition of eye lens membrane mainly consists of sphingolipids (sphingomyelin (SM) and dihydro-SM) and PLs (phosphatidylcholine (PC), phosphatidylserine (PS), and phosphatidylethanolamine (PE)) [81]. PLs consist of a “head” group and two fatty acid “tails” where the head denotes the type of PLs, for example, PC, PE, and PS. The fatty acids in the fiber cell membranes of the eye lens are predominantly palmitic (16:0, P) and oleic (18:1-cis, O) [81,82,83,84]. The lipid composition of the lens membrane varies between species [81,85,86,87,88]. Sphingolipids (mainly dihydro-SM) are dominant in humans, whereas PC is dominant in short-lived animals [81]. To mimic the lipid composition of the eye lens membrane, commonly used lipids are 1-palmitoyl-2-oleoyl-*sn*-glycero-3-phosphatidylcholine (POPC), 1-palmitoyl-2-oleoyl-*sn*-glycero-3-phosphatidylserine (POPS), and 1-palmitoyl-2-oleoyl-*sn*-glycero-3-phosphoethanolamine (POPE) and SM, which are commercially available. The primary binding site of α-crystallin in the eye lens’s fiber cell plasma membrane is intrinsic PLs and sphingolipids [89,90,91]. The observation of amplified binding of α-crystallin to the lens membrane when surface proteins are stripped by trypsin degradation [92] and using urea [66,93] further supported that the primary binding site of α-crystallin in the eye lens’s fiber cell plasma membrane is PLs and sphingolipids.

### 2.1. Nature of α-Crystallin Binding to Lipids (PLs and Sphingolipids) of the Lens Membrane

**Figure 2:**
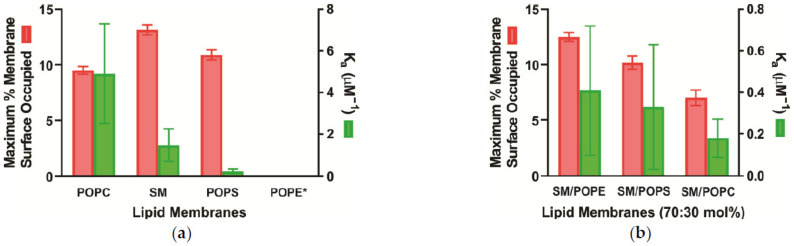
The maximum percentage of membrane surface occupied (MMSO) by α-crystallin and the binding affinity (K_a_) of α-crystallin binding to the lipid (PL and sphingolipid) membranes. (**a**) Individual lipid membranes, i.e., POPC, SM, POPS, and POPE* (where * represents the presence of 30 mol% POPC); (**b**) Two-component lipid membranes, i.e., SM/POPE, SM/POPS, and SM/POPC in 70:30 mol%. Redrawn with permission from [99,101], Copyright 2021, with permission from Taylor & Francis and Elsevier.

Many studies probed the interaction of α-crystallin with the lens membranes [89,90,93,94,95,96,97] and lipid vesicles [89,91,93,98,99,100,101,102]. Cobb and Petrash [98] conjugated α-crystallin with AlexaFluor350^TM^ fluorescent tag and studied the interaction of α-crystallin with SM membrane and PC membranes with a variety of acyl chain lengths and reported that the binding of α-crystallin to these membranes is non-saturable and not lipid-specific. However, the saturable binding of α-crystallin to the lipid vesicles has been reported earlier [89,91,100]. Ifeanyi and Takemoto et al. [91] also reported that α-crystallin binds to the individual PC, SM, and PE membrane in a saturable manner and in the same amount as with the intrinsic lens membranes. Tang et al. [102] used fluorescent probe NBD-PE that resides near the headgroup region of the membrane and observed saturable binding of α-crystallin in the SM membrane. The recent electron paramagnetic resonance (EPR) studies performed with cholesterol analog cholestane spin-label (CSL) that locate near the headgroup regions of the membrane [99,101] reported saturable binding of α-crystallin to POPC, POPS, and SM membranes and no binding of α-crystallin to the POPE membrane (Figure 2a). The EPR approach demonstrated the capacity to estimate the maximum percentage of membrane surface occupied (MMSO) by α-crystallin in different membranes where MMSO for SM and POPS membranes is larger in comparison to the POPC membrane (Figure 2a). The difference in the MMSO could be explained by fitting the saturation binding curve with the equation of specific binding with the Hill coefficient (h) using GraphPad Prism (San Diego, CA, USA), where h greater than 1 indicates that α-crystallin binds to the membrane with positive cooperativity, h less than 1 indicates that α-crystallin binds to the membrane with negative cooperativity, and h equal to 1 indicates that α-crystallin binds to the membrane with no cooperativity. Interestingly, h for SM and POPS membranes is greater than 1, whereas h for the POPC membrane is close to 1, indicating positive cooperativity for SM and POPS membranes and no cooperativity for the POPC membrane resulting in the difference in the MMSO (Figure 2a). The h for the SM membrane is higher than for the POPS membrane. This likely explains the larger MMSO for the SM membrane than for the POPS membrane. The detailed chemical explanation of the cooperativity and the mechanism of the difference in the cooperativity depending on the lipid type needs further investigation. Tang et al. [102] used the fluorescence approach and reported that a higher amount of α-crystallin binds to the SM membrane than with the PC membrane. If MMSO and binding affinity (K_a_) are compared with different lipid types, MMSO and K_a_ for different lipids are different, indicating that binding is lipid-specific (Figure 2a).

The MMSO for individual (Figure 2a) and two-component (Figure 2b) lipid membranes is comparable to the percentage of α-crystallin bound to the PL vesicles reported by Mulders et al. [96], where they labeled α-crystallin with [35S] methionine and incubated it with various concentrations of egg yolk lecithin vesicles (PC vesicles) and found ~10% α-crystallin bound to vesicles. The K_a_ values reported in Figure 2a,b for the individual and two-component lipid membranes are slightly different from those reported earlier [90,96,97], because these earlier studies used lens plasma membrane, including integral membrane proteins. The integral membrane proteins significantly influence the α-crystallin binding to the lens membranes, as discussed in Section 4.

Even with many studies, the nature of α-crystallin interaction with lipid vesicles is unclear. A few earlier studies indicated that the ionic interactions between α-crystallin and lipids influence binding [96,103]. The studies on α-crystallin binding to synthetic lipid membranes [66,93,100,102,104] and bovine lens lipid membranes [79,89,105] suggested that α-crystallin binds to lipid membranes noncovalently. α-crystallin has hydrophobic regions on its surface [96,106]. α-crystallin remains in the highly polydisperse oligomeric form [11,19], with the exchange of its subunits between oligomers [19,76,77,78]. Cobb and Petrash et al. [92] proposed that the interaction between α-crystallin and fiber cell plasma membrane of the eye lens membrane is hydrophobic, which occurs between the α-crystallin and hydrophobic fatty acid core of the membrane. A few earlier studies [90,102,107] indicated that the hydrophobic surface of the α-crystallin influences the binding of α-crystallin to the membranes. A study performed using the resonance energy transfer method suggested that α-crystallin preincubated at a higher temperature (65 °C) binds deep into the membrane [79], possibly due to exposure of more hydrophobic surfaces of α-crystallin when it is preincubated at a higher temperature. Recent EPR studies [99,101,108] and an earlier infrared spectroscopy study [109] reported that the polar headgroup of lipids strongly affects the α-crystallin binding to the membrane. Cobb and Petrash [98] proposed that the membrane’s surface is the only limiting factor; however, the lipid type, headgroups, and acyl chain length or saturation do not influence the interaction of α-crystallin with the membranes. Their conclusion is that the increase in α-crystallin binding to the in-vivo membrane is not a result of lipid changes but is likely due to the accumulation of high molecular weight forms of α-crystallin. The different MMSO and K_a_ values obtained (Figure 2a) for the PL membranes with the same hydrophobic fatty acid core (i.e., POPC, POPS, and POPE* membranes) imply that PL’s headgroup size and charge, hydrogen bonding between headgroups, and PL curvature influence the spacing between the headgroups and control the exposure of the hydrophobic core of the membrane. This likely modulates the possible hydrophobic binding between α-crystallin and membrane [101]. Although Cobb and Petrash [98] reported that acyl chain length or saturation of lipids do not influence the binding of α-crystallin to lipid membranes, the likely hydrophobic binding of α-crystallin to the lipid membranes warrants more studies along this line of research for a deeper understanding. Since the MMSO by α-crystallin is small for the individual lipid membranes (Figure 2a) and two-component lipid membranes (Figure 2b), we suggest that only the hydrophobic regions exposed on the outer surface of α-crystallin oligomers contribute to the binding. Due to the larger size of the α-crystallin oligomer, the steric hindrance between bound α-crystallin oligomers after certain binding is achieved may also contribute to smaller MMSO.

The lipid (PL and sphingolipid) composition in the fiber cell plasma membrane of the eye lens changes with age and cataract [2,82,110,111,112,113,114,115], among species [81,110,111,116], and location in the lens [4,81,82,113,117,118]. The MMSO by α-crystallin and the K_a_ values are different for different lipid membranes (Figure 2a). Additionally, for two-component lipid membranes (Figure 2b), the change in lipid composition changes the MMSO by α-crystallin and the K_a_ values. Based on these data (Figure 2a,b), we suggest that the change in lipid composition may be the translocation event that promotes the translocation of α-crystallin from the lens cytoplasm to the fiber cell plasma membranes. This is further supported by the observations that the binding of α-crystallin to the lens membranes increases with age and cataract formation [59,60,61,62,64,65], as the lipid composition changes dramatically with age and cataract [2,82,110,111,112,113,114,115].

### 2.2. Binding of α-Crystallin to the Lens Lipid Membrane Changes the Membrane’s Physical Properties

**Figure 3:**
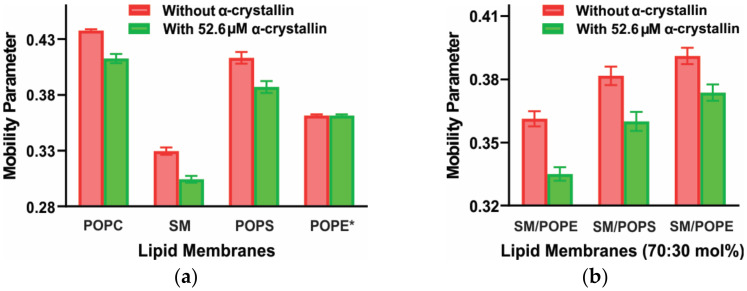
The decrease in the mobility parameter of the lipid (PL and sphingolipid) membranes after α-crystallin binding. (**a**) Individual lipid membranes, i.e., POPC, SM, POPS, and POPE* (where * represents the presence of 30 mol% POPC). (**b**) Two-component lipid membranes, i.e., SM/POPE, SM/POPS, and SM/POPE in 70:30 mol%. The mobility parameter decreases for the individual lipid membranes (POPC, SM, and POPS) and two-component lipid membranes (SM/POPE, SM/POPS, and SM/POPE in 70:30 mol%) with an increase in α-crystallin concentration, representing the decrease in the mobility of the membranes near the headgroup regions due to the increase in the binding of α-crystallin to these membranes. The mobility parameter for the POPE* membrane does not decrease in the presence of α-crystallin, because α-crystallin does not bind to the POPE* membrane. Redrawn with permission from [99,101], Copyright 2021, with permission from Taylor & Francis and Elsevier.

The association of α-crystallin to the lens lipid membrane changes the membrane’s physical properties, such as the mobility parameter and maximum splitting [99,101,108,119,120,121,122]. The mobility parameter gives the orientational and rotational dynamics of the cholesterol analog spin-label (CSL) in the membrane [119,121,122]. The maximum splitting is a parameter related to the order parameter that gives the amplitude of the wobbling motion of the long axis of the CSL spin-label in the membrane [120,121,122]. Figure 3a,b show the decrease in the mobility parameter of the individual lipid membranes (POPC, SM, and POPS) and two-component lipid membranes (SM/POPE, SM/POPS, and SM/POPC), respectively, in the presence of α-crystallin, indicating that those membranes become less mobile near the headgroup regions after α-crystallin binding. Using the fluorophore NBD-PE, which partitions near the membrane headgroup regions, Borchman and Tang [89] found a similar decrease in the mobility of the headgroups of bovine lens lipid vesicles upon α-crystallin binding. The higher the MMSO or the amount of α-crystallin bound to the membranes, the greater the decrease in the mobility parameter.

The maximum splitting of the SM and the SM/POPE in 70:30 mol% membranes increased with an increase in α-crystallin concentration [101], implying that these membranes become more ordered near the headgroup regions after the binding of α-crystallin. It is reported that α-crystallin binding to the protein-free membranes depends on the hydrocarbon chain order [102]. With age, an increase in the amount of SM and a decrease in the amount of PC has been observed [82,123]. The highest order near the surface of the SM membrane and lowest order near the surface of the POPC membrane (Figure 4B in [101]) support the observation of increased order of the lens lipids with aging [117,124].

## 3. Role of Cholesterol (Chol) on the α-Crystallin Binding to the Lens Membrane

### 3.1. Extremely High Chol Content Forming Cholesterol Bilayer Domains (CBDs) within the Lens Membrane

Chol is one of the major components of the eye lens’s fiber cell plasma membrane [3,4,5]. Compared to other eukaryotic membranes, the eye lens’s fiber cell plasma membrane contains extremely high Chol content [125]. The Chol/lipid molar ratio in the chicken, cow, human, and whale’s eye lens’s fiber cell plasma membrane ranges from 1 to 10 [85,111]. Whale’s eye lens membrane has the highest Chol/lipid molar ratio of 10 [111]. Humans have a Chol/lipid molar ratio as high as 1.8 in the cortical membranes and 4.4 in the nuclear membranes [84,113,115,126]. The Chol/lipid molar ratio in the human eye lens membrane increases with age [113,115,126] and decreases with the cataract formation [127].

With an increase in Chol concentration in the Chol-containing PL membranes, Chol saturates the membrane with the formation of phospholipid cholesterol domain (PCD) [113,127,128,129]. With further increase in Chol concentration, CBDs start to form, which coexist with surrounding PCD [113,127,128,129]. CBDs were observed in the model membranes [121,122,130,131,132,133,134], lens lipid membranes [113,118,127,128,129], and the intact cortical as well as nuclear membranes isolated from the human donors who were 40, 46, and 53 years old [135]. The bulk physical properties of lens lipid membranes remain consistent and independent of changes in lipid composition due to saturating Chol content [125,136,137]. Thus, CBDs help maintain lens membrane homeostasis by providing the buffering capacity for Chol concentration in the surrounding lipid bilayer, keeping it at a constant saturation level. This is particularly important for human lenses because of the longest life span and the most pronounced age-related changes in lens lipid composition compared to other mammalian lenses [138]. Other functions of Chol include the formation of a hydrophobic barrier and altered rigidity across the membrane lipids [121,139,140]. When the Chol content exceeds the Chol solubility threshold, Chol crystals form, presumably outside the membrane [130,141]. Since the Chol level increases with age, it exceeds the Chol solubility threshold, with Chol crystals forming outside the lens membranes [126].

### 3.2. Chol and CBDs Inhibit the Binding of α-Crystallin to the Membranes Made of Eye Lens Lipids (PLs and Sphingolipids)

**Figure 4:**
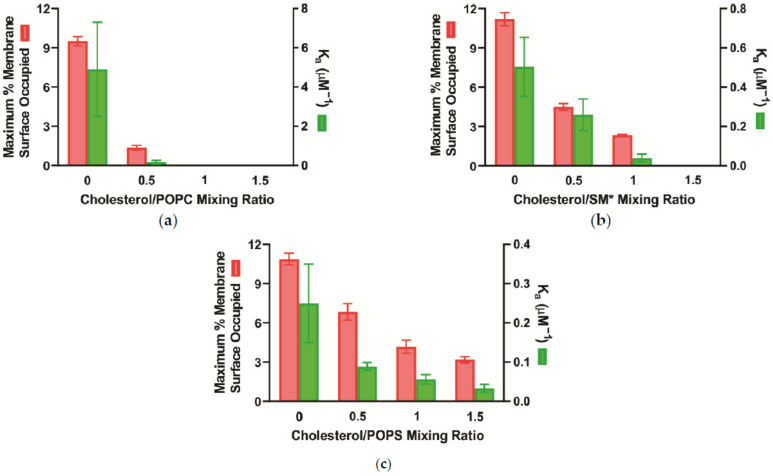
The maximum percentage of membrane surface occupied (MMSO) by α-crystallin and the binding affinity (K_a_) of α-crystallin binding to the cholesterol/lipid (Chol/lipid) membranes at Chol/lipid mixing ratios of 0, 0.5, 1, and 1.5. (**a**) Chol/POPC membranes; (**b**) Chol/SM* membranes, where * represents the presence of 20 mol% POPS; (**c**) Chol/POPS membranes. With an increase in Chol/lipid mixing ratio for the Chol/POPC, Chol/SM*, and Chol/POPS membranes, both the MMSO by α-crystallin and K_a_ decrease, indicating that Chol inhibits the binding of α-crystallin to these membranes. Redrawn with permission from [99,101,108], Copyright 2021, with permission from Taylor & Francis and Elsevier.

The role of Chol on α-crystallin binding to the lens membrane and lipid vesicles has not gained sufficient attention. Cobb and Petrash [98] used human recombinant AlexaFluor350^TM^-conjugated α-crystallin with the PC and SM membranes with and without 40 mol% Chol and reported no significant difference in the binding of α-crystallin to PC and SM membrane with and without Chol. However, Tang et al. [102] used distearoyl-phosphatidylcholine (DSPC), SM, and egg-phosphatidylcholine (egg-PC) membranes with Chol/lipid weight ratio up to 1.0 (~64 mol% Chol) and performed fluorescence studies using fluorophore NBD-PE, which resides in the polar headgroup regions of the membrane, and reported a significant decrease in the binding of α-crystallin with the DSPC and SM membranes with Chol. Additionally, Tang et al. [102] reported a slight increase in the binding of α-crystallin to the egg-PC in the presence of Chol. Moreover, a few studies reported that Chol inhibits the binding of α-crystallin to the lipid vesicles [89,91]. The recent study [108] performed using the EPR spin-labeling method shows that an increase in Chol content in POPC, SM*, and POPS membranes decreases the α-crystallin binding to the membranes; however, the decrease in the binding is different for different lipid types (Figure 4).

The EPR spin-labeling studies reported that hydrophobicity near the surface of the Chol/POPC [122], Chol/SM [131], and Chol/POPS [132] membranes decreases with an increase in Chol content. Interestingly, the MMSO and K_a_ decrease with an increase in Chol concentration for the Chol/POPC, Chol/SM*, and Chol/POPS membranes (Figure 4). These results indicate that interaction between α-crystallin and Chol/lipid membranes is likely hydrophobic. The small MMSO for the Chol/lipid membranes (Figure 4) further supports that only the hydrophobic regions exposed on the outer surface of α-crystallin oligomers contribute to the binding, with the possibility that the steric hindrance between bound α-crystallin oligomers after certain binding is achieved also contribute to the smaller MMSO. The different MMSO and K_a_ values of different Chol/PL membranes with the same hydrophobic core (Figure 4) imply that the likely hydrophobic binding of α-crystallin and the Chol/PL membranes is modulated by the lipid headgroup’s size and charge, hydrogen bonding between the lipid’s headgroups, and PL curvature. Moreover, the degree to which the MMSO and K_a_ decrease differently for different Chol/lipid membranes (Figure 4) is likely due to the strength of Chol’s interactions with the different lipids. An important point to note is that, irrespective of the lipid type, the data (Figure 4) show that an increase in Chol concentration decreases the MMSO and K_a_, implying that Chol and CBDs play a positive physiological role by inhibiting the binding of α-crystallin to the lens membranes and possibly protecting against cataract formation.

If MMSO and binding affinity (K_a_) are compared between different lipid types, MMSO and K_a_ for different Chol/lipid membranes are different, indicating that binding is lipid-specific (Figure 4). Several studies performed earlier in various research laboratories [89,91,100] and a recent EPR study performed in our laboratory [108] reported that the α-crystallin binding to the Chol/lipid membranes is saturable.

The Chol concentration in the fiber cell plasma membrane of the lens changes with age and cataract [113,115,126,127,142], among species [85,111], and location in the lens [84,113,115,126]. The Chol/lipid molar ratio increases with age and decreases with cataract formation [113,115,126,127,142]. With different Chol content among species, different cataract onset ages have been observed. Humans have a Chol/lipid molar ratio of up to 4 [81] and generally develop cataracts at 60 years of age [111,116], but whales have a Chol/lipid molar ratio of 10 and do not develop cataracts until up to 200 years of age [111]. Most probably, the high amount of Chol in a whale’s eye lens protects α-crystallin binding with its lens membranes, protecting them from cataract formation. The Chol/lipid molar ratio and CBDs’ size on the cortical and nuclear membranes extracted from human donors 61–70 years old were smaller in cataractous lenses than in clear lenses [127]. Moreover, CBDs’ size increased with an increase in Chol content in the lens membrane [113]. These previous observations [113,127], including the recent observations (Figure 4), imply that the larger Chol/lipid molar ratio and CBDs’ size decreases the binding of α-crystallin to the lens membranes, possibly protecting from cataract formation.

### 3.3. Binding of α-Crystallin to the Chol/Lipid Membrane Changes the Membrane’s Physical Properties

**Figure 5:**
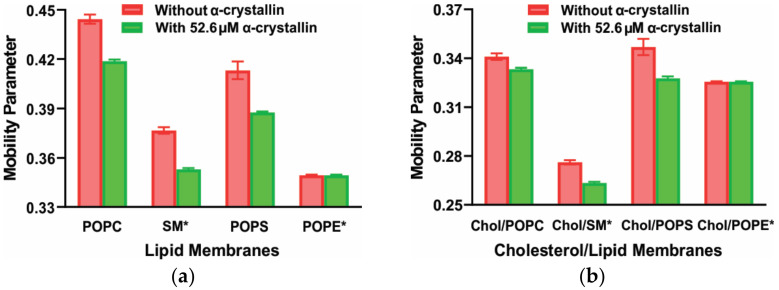
Mobility parameter of lipid (PL and sphingolipid) membranes with and without α-crystallin. (**a**) Individual lipid membranes, i.e., POPC, SM*, POPS, and POPE* (where * represents the presence of 20 mol% POPS); (**b**) Chol/POPC, Chol/SM*, Chol/POPS, and Chol/POPE* membranes with Chol/lipid mixing ratio of 0.3. The mobility parameter decreases for the individual lipid membranes (POPC, SM*, and POPS) and Chol/lipid membranes (Chol/POPC, Chol/SM*, and Chol/POPS) with an increase in α-crystallin concentration, indicating the decrease in the mobility of the membranes near the headgroup regions due to the binding of α-crystallin to these membranes. The mobility parameter for the POPE* and Chol/POPE* with Chol/POPE* mixing ratio of 0.3 membranes does not decrease in the presence of α-crystallin, because α-crystallin does not bind to these membranes. Redrawn with permission from [99,101,108], Copyright 2021, with permission from Taylor & Francis and Elsevier.

The mobility parameter of the lipids (Figure 5a) and Chol/lipid (Figure 5b) membranes decrease with an increase in α-crystallin concentration, implying that these membranes become less mobile near the headgroup regions after the α-crystallin binding. Borchman and Tang et al. [89] also found a similar decrease in the mobility of the headgroups of bovine lens lipid vesicles upon α-crystallin binding.

An increase in Chol concentration in each Chol/lipid membrane decreases the membrane’s mobility near the headgroup regions and antagonizes the ability of α-crystallin to decrease the mobility near the membrane’s headgroup regions (Figure 5).

An increase in Chol concentration in each Chol/lipid membrane increases the maximum splitting of the membrane, indicating that Chol increases the membrane’s order near the headgroup regions [108].

### 3.4. Hight Chol Content and Lens Transparency

**Figure 6:**
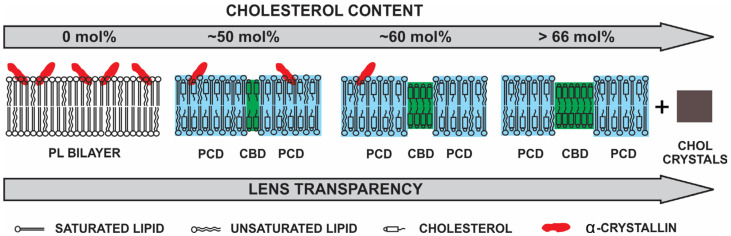
Schematic drawing showing the decreased α-crystallin binding to the lipid membrane with increased Chol content. An increase in Chol content saturates the membrane, forming the phospholipid cholesterol domain (PCD). With a further increase in Chol content, cholesterol bilayer domains (CBDs) coexist with the PCD. CBDs start to form at 50, 48, 46, and 33 mol% Chol within the PC, SM, PS, and PE membranes, respectively [130]. CBDs are shown in green color in the schematics. The CBD’s size increases with an increase in Chol content, and at above 66 mol% Chol, Chol crystals form, presumably outside the bilayer [113]. The current study’s findings [108] suggest that the binding of α-crystallin to the lens lipid membrane decreases with an increase in Chol content, and no α-crystallin binds to the lipid membrane above likely ~60 mol% Chol in the membrane. The real size of the α-crystallin oligomer (red) is much larger than represented in this figure. The decrease in the binding of α-crystallin to the Chol/lipid membranes made with increasing Chol content represents the decrease in light scattering and increase in lens transparency. Adapted from [108], Copyright 2021, with permission from Elsevier.

Based on our results of α-crystallin binding to the Chol/lipid membranes at different Chol/lipid mixing ratios (Figure 4), Figure 6 shows the schematic drawing showing the decrease in the binding of α-crystallin to the Chol/lipid membrane with an increase in Chol concentration. The decrease in the binding of α-crystallin to the lens membranes increases the lens transparency (Figure 6). Figure 6 shows the penetration of a small portion of α-crystallin oligomer into the membrane. A few studies [2,66,79] reported that denatured α-crystallin binds deep into the membrane. How much α-crystallin penetrates the membrane and what factors affect it are open questions and need further investigation. Note that Figure 6 shows the binding of α-crystallin to the lipid membrane without the integral membrane proteins, the high concentration of which remains in the lens plasma membrane. Section 4 explains the effect of the integral membrane proteins on α-crystallin binding to the lens plasma membrane.

### 3.5. Lipid Peroxidation and Cataract Formation

Regardless of a relatively low oxygen level in the eye lens, photo and chemical oxidation of lipids can occur and affect it [2]. The oxidation of lipids in the human lens with age has been studied [143,144,145,146,147]. The major secondary product of lipid oxidation is malondialdehyde (MDA), which increases in the human lens with age [147] and cataract formation [143,146,147,148,149,150,151,152]. Both the clear and cataractous lenses consist of lipid oxidation products [143,144,145,146,148,149,152,153]. More than 40% of the lens PLs degrade, forming oxidation products in the human lens over the lifetime [2]. With a cataract formation, even more amounts of PLs degrade in the human lens [2,112]. There is no turnover of lipids in the human lens for the entire lifespan [154]. The oxidative damage in the lens accumulates, which changes the crystallin’s structure, resulting in light scattering [111]. A proposition is that the α-crystallin binding to the lens membrane acts as a seed for lipid oxidation and other protein’s binding to the membrane, resulting in protein aggregation and light scattering [79]. Many studies have proclaimed that lipid oxidation may initiate the pathogenesis of human cataracts [143,146,147,148,149,150,151,152].

A hypothesis is that the oxidation of glycerophospholipids is a primary cause of change in lipid composition over age and cataract formation [111]. With age, sphingolipids increase, and glycerophospholipids decrease in the human lens [112]. Moreover, with cataract formation, the amounts of both the sphingolipids and glycerophospholipids decrease [2,112]. However, the decrease in sphingolipids is much less in extent [112]. Lipid oxidation is less favorable with the decrease in the number of lipid double bonds [155]. Sphingolipids are 3–4 times more saturated than glycerophospholipids [156,157]. Therefore, sphingolipids resist oxidation more effectively than glycerophospholipids [155,158,159,160,161,162]. Moreover, the degree of oxidation of polyunsaturated PC is less favorable with SM in the membrane than without SM [158], implying that lens sphingolipids hinder the oxidation of unsaturated glycerophospholipids. Human lens membranes have a high concentration of sphingolipids (SM plus dihydrosphingomyelin (DHSM)) [81], which provide resistance to oxidation and allow the lens membranes to remain clear relatively longer than in the case of many other species [117]. In the whale’s eye lens membrane, the dominant amount of DHSM (100% DHSM) helps provide resistance to oxidation and allows the lens membrane to remain clear even up to 200 years of age [111].

The lipid peroxidation induces cholesterol domain formation at lower Chol content in the Chol-containing PL membranes [163,164]. The peroxidation of polyunsaturated PL [165] in the Chol-containing PL membranes decreases the Chol concentration at which CBDs and Chol crystals start to form. Chol crystals form presumably outside the membrane bilayer [130,141]. The formation of CBDs and Chol crystals at the lower Chol content decreases the overall Chol content within the membrane. Furthermore, data from literature [102,108] indicate that the higher the Chol concentration in the Chol-containing lipid membrane, the lower the α-crystallin binding to the membrane. These observations suggest that lipid oxidation decreases the Chol content within the lens membrane, which likely increases the binding of α-crystallin to the lens membranes and possibly promotes cataract formation.

Lipid oxidation plays a governing role in increasing senescent membranes’ order in many systems [166]. The oxidation has a dual effect on SM’s order [167]. The hydrocarbon chain order of SM increased with mild oxidation and decreased with strong oxidation [167]. Other studies have reported that lipid oxidation orders the hydrophobic chains [148,168,169,170,171,172,173,174,175,176]. It is not clear if the increased order of hydrophobic chains due to the lipid oxidation correlates with the binding of α-crystallin to the membrane and cataract formation.

## 4. Interaction of α-Crystallin with the Lens Integral Membrane Proteins

The fiber cell plasma membrane of the eye lens consists of two major types of integral membrane proteins, namely aquaporin-0 (AQP0), also known as major intrinsic protein (MIP26) [6,7,8], and connexins (Cx43, Cx46, and Cx50) [9]. The transport and communication between the lens’s fiber cells are allowed by the thin and gap junctions [177]. AQP0 or MIP26, which accounts for more than 60% of the lens’s membrane protein [178], makes water channels [179] also known as the thin junction. The combination of two tetramers of MIP26 from the neighboring fiber cells forms a thin junction [180]. Thin junctions control the transport of water and some neutral solutes [181]. The mutations in human MIP26 resulting in autosomal dominant polymorphic and lamellar cataract formation [182,183,184] point out the vital role of MIP26 in lens transparency. These pathological mutations in MIP26 possibly perturb water content in the lens fiber cells and affect cell-to-cell adherence [182]. Gap junctions, also known as membrane channels, are made of three kinds of connexins, Cx43, Cx46, and Cx50 [9,185]. Cx46 is found mainly in the cortex and the outer nuclear layers, and Cx50 is found mainly in the nuclear core [186,187,188]. Six connexins form a connexon, and two connexons from neighboring cells form a gap junction. Gap junctions control the exchange of ions and small metabolites (amino acids, glucose) between the lens cells [185,189].

**Figure 7:**
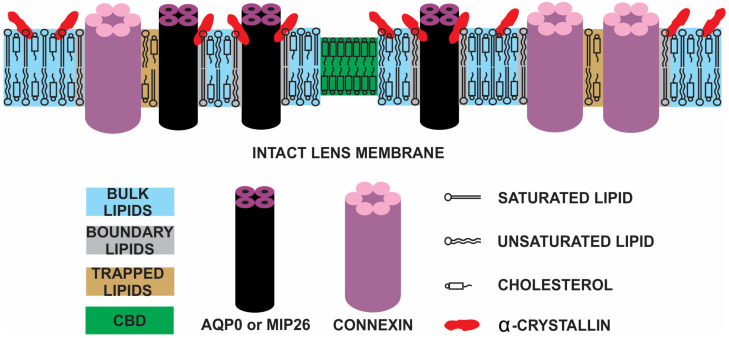
Schematic drawing showing an intact human lens membrane. The integral membrane proteins aquaporin-0 (AQP0) or MIP26 and connexins (Cx43, Cx46, and Cx50) remain embedded into the lens membrane. α-crystallin (red) binds to the lipids (PLs and sphingolipids) and MIP26, most possibly via the hydrophobic core of the lipids [92] and MIP26 [96]. The recent data for the lipids [99,101] and Chol/lipid membranes [108] suggest that hydrophobic regions exposed on the outer surface of α-crystallin oligomers penetrate the membrane to bind to lipids and MIP26. The real size of the α-crystallin oligomer is much larger than represented in this figure. MIP26 forms thin junctions, which control the transport of water and some neutral solutes between lens fiber cells. Connexins form gap junctions, which control the exchange of ions and small metabolites between the lens fiber cells. Blocking these membrane pores by α-crystallin binding to the membranes creates a barrier to the small molecules leading to nuclear cataract development [64]. Four kinds of lipid environments exist in the human lens membrane, such as bulk lipids (blue), boundary lipids (grey), trapped lipids (gold), and cholesterol bilayer domains (CBDs) (green) [135,196,197]. MIP26 and connexins likely induce the boundary and trapped lipids’ formation [8,198]. The water permeability through the thin junctions depends on its lipid environment [199]. The Chol content in the human lens membrane is extremely high, forming CBDs within the membrane [113,135]. Note that Chol is excluded from the boundary lipids [200,201].

A few studies using isolated crystallins and MIP26 suggested that α-crystallin binds to MIP26 [96,190,191]. However, a few other studies claim that α-crystallin mainly binds to lipids [66,89,90,91,93,98]. Mulders et al. [96] used PL vesicles with MIP26 reconstitution and showed that α-crystallin binding depends on the presence of MIP26. Trypsin pretreatment, which converts MIP26 to MIP22, or preincubation of vesicles with antibodies against MIP26, did not influence the binding of α-crystallin [96], suggesting that the amino-terminal cytoplasmic fragment [192] is not involved in the association. This agrees with the earlier observation that α-crystallin is still present in protease-treated membranes, which contain MIP22 instead of MIP26 [193]. Therefore, the possible means of α-crystallin interaction with MIP26 is through the hydrophobic core of the MIP26, which implies that part of α-crystallin penetrates the membrane, as suggested by Bloemendal et al. [193]. How much part of the α-crystallin oligomer penetrates the membrane is an open question and needs further investigation. Based on our observations of α-crystallin binding to the lipid and Chol/lipid membranes reported in Section 2.1 and Section 3.2, respectively, we suggest that the hydrophobic regions exposed on the outer surface of the α-crystallin oligomers penetrate the membrane. However, denatured α-crystallin binds deep into the membrane [2,66,79]. Figure 7 shows the penetration of α-crystallin into the membrane to bind to the hydrophobic regions of MIP26 and lipids. The real size of the α-crystallin oligomer (red) is much larger than represented in Figure 7. A confocal fluorescence resonance energy transfer (FRET) microscopy study [191] using MIP26 tagged with a green fluorescence protein (GFP) as a donor and αA-, αB-, βB2-, or γC-crystallin tagged with red fluorescence protein (RFP) as an acceptor shows that αA-, αB-, βB2-, and γC-crystallin interact with the MIP26. However, the binding between MIP26 and both the αA- and αB-crystallin was considerably higher [191]. αA- and αB-crystallin have greater hydrophobicity than βB2- and γC-crystallin [194]. Therefore, considerably higher binding of αA- and αB-crystallin to MIP26 than βB2- and γC-crystallin to MIP26 [191] supports the hydrophobic interaction between α-crystallin and MIP26. α-crystallin undergoes various mutations and PTMs with age and cataract formation (see Section 5). Additionally, MIP26 undergo extensive PTMs with age [195]. There may be some PTMs in α-crystallin and/or MIP26 that increase the association of α-crystallin with MIP26 in lens membrane, following light scattering and cataract formation. Therefore, investigations exploring PTMs in α-crystallin and/or MIP26 responsible for the association of α-crystallin with MIP226 are crucial.

A study using the saturation recovery (SR) EPR method demonstrated that the oxygen transport parameter (OTP) decreases with an increase in α-crystallin binding to the PC membrane [99], indicating that binding of α-crystallin to the membrane forms a barrier to oxygen transport. Although the study [99] used an individual PL membrane without integral membrane proteins, the result helps to speculate that the binding of α-crystallin to the fiber cell plasma membrane of the eye lens creates a barrier, in agreement with the barrier hypothesis proposed earlier [30,63,64]. The barrier may block the membrane pores formed by MIP26 and connexins, producing an oxidative condition in the lens, leading to nuclear cataract development [30,63,64].

The water permeability through the AQP0 (MIP26) decreases with increasing Chol and SM content [199], implying that the bilayer lipid composition regulates water permeability. Since the concentration of Chol and SM is high in the lens nucleus compared to the cortex [83,84,117,202,203,204], AQP0 permeability would be significantly less in the lens nucleus than in the lens cortex [199]. As the increasing Chol content decreases binding of α-crystallin to the Chol/lipid membranes and α-crystallin binding depends on the type of lipid in the membrane [99,101,108], the Chol content and change in lipid composition likely modulate the binding of α-crystallin to the MIP26. However, more investigations are necessary along this line of research.

Gap junction, which transports metabolites and ions between lens cells [189], is vital for maintaining lens homeostasis of inner fiber cells. A study [189] using double knockout mice characterizing lens phenotypes demonstrated that the absence of gap junctions between lens fiber cells displayed severe cataracts resulting from cell swelling and degeneration of inner fiber cells. This result suggests that a loss of gap junction leads to cataract formation. This result further supports the barrier hypothesis that the association of α-crystallin to the lens membrane may block the transport of metabolites and ions through gap junctions, forming nuclear cataracts [30,63,64]. Whether α-crystallin directly binds to the connexins blocking the membrane pores or not still needs investigation.

## 5. Mutations and Post-Translational Modifications (PTMs) of α-Crystallin, Resulting in Association of α-Crystallin to the Fiber Cell Plasma Membranes and Cataract Formation

### 5.1. Mutations in α-Crystallin That Cause Association of α-Crystallin to Lens Membranes

The studies using in-vitro assays and site-directed mutagenesis have suggested that α-crystallin usually is stable and can tolerate the substitution of its many amino acids in its primary structure [205,206]. Several mutations leading to cataract formation have been reported [207,208,209,210]. A missense mutation of the arginine 116 residue to cysteine (R116C) in αA-crystallin decreases the chaperone-like activity of α-crystallin approximately four-fold, reduces the ability to exchange α-crystallin’s subunits four-fold, and increases the membrane-binding capacity 10-fold [208]. Such reduced chaperone-like activity and increased membrane-binding capacity of α-crystallin cause a zonular central nuclear cataract [208,211]. Moreover, mutation R116C in αA-crystallin showed less binding with actin, which is necessary for the normal differentiation of lens cells, compared to wild-type αA-crystallin [209]. This implies that the R116C mutation causes decreased chaperone activity and increased aggregation of αA-crystallin [209]. The reverse-phase high-performance liquid chromatography study suggested that mutation of the arginine 116 residue to histidine (R116H) in αA-crystallin increased hydrophobicity of the αA-crystallin compared to that of wild-type αA-crystallin [212]. The DTT (DL-dithiothreitol)-induced insulin aggregation assay showed the loss of chaperone activity of the αA-crystallin due to R116H mutation [212]. Furthermore, fast protein liquid chromatography (FPLC) purification showed that mutation R116H to αA-crystallin increased its binding affinity to lysozyme [212]. A missense mutation of the arginine 120 to glycine (R120G) in αB-crystallin [213,214,215] causes a desmin-related myopathy and congenital cataracts, because this mutation alters protein–protein interaction, with an increase in its cytoplasmic aggregation. The analysis of recombinant αA- and αB-crystallin containing these mutations has shown marked changes in the α-crystallin’s chaperone function [10]. Pras et al. [216] reported that a nonsense mutation of the glycine residue to alanine in αA-crystallin caused autosomal recessive cataracts in an inbred Jewish Persian family, resulting in the formation of the premature stop codon (W9X). Moreover, a dominant valine 124 to glutamic acid (V124E) mutation in mouse αA-crystallin has been reported [217]. A dominant congenital posterior polar cataract in humans caused by a mutation in the αB-crystallin gene resulted in 35 totally different residues at the C-terminus of the αB-crystallin gene [218]. The site-directed mutagenesis studies showed phenylalanine 71 (F71) in αA-crystallin is necessary for its chaperone activity [219,220]. A missense mutation of F71 to leucine (i.e., F71L) in αA-crystallin decreases the chaperone activity of αA-crystallin and induces age-related cataracts in humans [221].

Grosas and Carver et al. [210] discussed several other α-crystallin mutations leading to cataract formation. We speculate that future investigations will discover additional mutations leading to cataract formation and progression. The abnormal α-crystallin caused by mutations may precipitate in the lens, cause the precipitation of other lens proteins, and associate with the lens membranes, causing the scattering of light and cataract formation [20]. The binding of α-crystallin to the lens membranes depends on the surface area of the hydrophobic regions exposed on the outer surface of α-crystallin oligomer [99,101,108]. Different mutations may cause distinct changes to the α-crystallin’s structure and the surface area of the hydrophobic regions exposed on the surface of its oligomeric form. This is why different mutations likely have a different extent of α-crystallin association with the fiber cell plasma membrane. More research on identifying which particular mutation of α-crystallin increases or decreases its association with the lens membrane is an open area of investigation and may contribute to a better understanding of cataract progression and development.

### 5.2. Post-Translations Modifications (PTMs) in α-Crystallin

Various studies of lens proteins have shown that PTMs may significantly affect the chaperone-like activity of α-crystallin [222,223,224,225,226]. PTMs induce conformational changes in α-crystallin, leading to possible changes in its interaction with the other proteins in the lens, damaging its chaperone functions [227]. The changes in α-crystallin caused by PTMs likely influence its association with the lens membranes; however, detailed studies are necessary to understand it clearly. Most cataracts are not congenital but develop with mutations and PTMs of α-crystallin during aging [228]. Several types of PTMs on α-crystallin have been reported to date, e.g., deamidation [229,230,231,232,233], phosphorylation [223,227,231,232,233,234,235,236], isomerization [237,238], acetylation [233,239,240], glycation [241,242,243], proteolytic cleavage [231,232,233,235,244,245], and oxidation [20,246,247,248]. Here, we discuss these common PTMs, focusing on chemical mechanisms founding these PTMs and their implications in decreasing the chaperone activity of α-crystallin, causing cataract formation.

#### 5.2.1. Deamidation

Deamidation is a chemical reaction where the amide group in the side chain of the amino acids asparagine (Asp) or glutamine (Glu) is removed or converted to carboxylic acid. Generally, Asp changes to aspartic acid, and Glu changes to glutamic acid. The net chemical change caused by deamidation is an addition of the water group and removal of the ammonia group, which results in one Da mass increase. Normally, deamidation events in lens proteins are harmful effects of aging and exposure to UV radiation [249,250]. Many different PTMs have been observed in rat lenses using proteomic analysis; however, deamidation of Asp and Glu amino acids was the most common [26]. Deamidation of Asp 123 of αA crystallin caused a significant decrease in its chaperon activity [229]. A study [231] investigating the α-crystallin of the water-insoluble but urea-soluble portion of 45-year-old normal lenses hypothesized that the deamidation of various Asp and Glu amino acids of α-crystallin makes α-crystallin water-insoluble, following conformational changes guiding the formation of the intramolecular disulfide bond between cysteine (Cys) residues of αA-crystallin. Deamidation occurs in all the crystallins in the older lenses; however, the extent of deamidation depends on individual polypeptides [251]. Deamidation of ~45% of Asp 101 in human αA-crystallin occurs in the early 30 years of age [230]. An additional ~5% deamidation of Asp 101 in human αA-crystallin occurs from 30 to 68 years of age [230]. Deamidation is more common in insoluble proteins than soluble proteins from the same lenses [252], indicating that deamidation may significantly contribute to the denaturation and aggregation of proteins. However, to what degree deamidation at any specific site contributes to the formation of cataracts is still an open question [238,253].

#### 5.2.2. Phosphorylation

Phosphorylation is a reversible PTM of proteins, where the amino acid residue is phosphorylated by the protein kinase with the addition of a covalently bound phosphate group. Phosphorylation of serine 45 (Ser 45) and Ser 122 in αA-crystallin occurs, with Ser 122 phosphorylation being the most common [234]. Phosphorylation of Ser 19, Ser 45, and Ser 59 in αB-crystallin occurs, with Ser 59 phosphorylation being the most common [254]. Phosphorylation changes the protein’s surface charge, accompanied by conformational changes, which may change the protein–protein interaction and reduce the chaperone function [227]. Chiappori et al. [255] modeled dimers and hexamers from the 24-meric structure of αB-crystallin (PDB id: 2YGD) and performed the molecular dynamics (MD) simulation studies with all Ser 45/Ser 59 phosphorylated and all non-phosphorylated forms. Their simulation studies [255] suggest that Ser 59 is a key residue for regulating the multimeric conformation of αB-crystallin. Kim et al. [236] reported that in diabetic OLETF rats, increased α-crystallin expression corresponds with increased phosphorylation on Ser 45 and Ser 59 of αB-crystallin, which negatively modulates the α-crystallin’s chaperone function. The mono-phosphorylation of αB-crystallin decreased α-crystallin’s chaperone-like activity by approximately 30% [223]. Phosphorylation increases with age and can affect the α-crystallin’s chaperone function [227,256]. The phosphorylation of Ser 122 of αA-crystallin does not occur during the aging process but is a developmentally controlled event in the human lens [234]. Furthermore, it is reported that the phosphorylation of α-crystallin does not change its binding to the lipid vesicles [98].

#### 5.2.3. Isomerization

Isomerization is a process in which a molecule or a molecular fragment converts to an isomer with a different chemical structure. Isomerization is one of the major PTMs of proteins, which could occur spontaneously and non-enzymatically, affecting the structure and functions of proteins. Isomerization of Asp 58, Asp 91 or Asp 92, and Asp 151 residues in αA-crystallin may cause aggregation of proteins to HMWCs, ultimately reducing chaperone activity and leading to cataract formation [237]. A study [238] performed using combined techniques, such as synthetic peptide mimics, enzyme assays, MD simulations, and native mass spectrometry experiments, reported that isomerization of Asp 62 in αB-crystallin alters phosphorylation of Ser 59 in αB-crystallin. Additionally, isomerization of Asp 109 in αB-crystallin disrupted a significant salt-bridge between Asp 109 and R 120 in αB-crystallin, introducing profound changes in protein structure [238].

#### 5.2.4. Acetylation

Acetylation is an organic esterification reaction with acetic acid. It is a reversible PTM, which introduces the acetyl functional group into the protein. Lysine 70 (K 70) and K 99 in αA-crystallin and K 92 and K 166 in αB-crystallin can be acetylated in the human lens [240]. The acetylation mimic of αA-crystallin by replacing K 70 with glutamine (K70Q) increased this protein’s chaperone function [240]. The suggestion is that acetylation of K 70 of αA-crystallin may affect its conformation and intermolecular interactions, consequently altering the solubility and chaperone function of αA-crystallin [233].

#### 5.2.5. Glycation

Glycation is a covalent attachment of sugar to a protein. The common sugars that participate in glycation are glucose, fructose, and their derivatives. The glycation of α-crystallin was observed in the human lens and a diabetic rat [241,242,243], resulting in an increase in size of the α-crystallin aggregates [243], leading to its decreased chaperone activity.

#### 5.2.6. Proteolytic Cleavage

Proteolytic cleavage is fundamentally the breaking of the bond between peptides in a protein. Various sites of crystallin cleavage have been reported [250,257,258]. The C-terminus of αA-crystallin is the most predominant age-dependent cleavage site, which results in the loss of chaperone activity of this protein [245]. The peptides produced from the cleavage may themselves have crucial biological significance. For example, α-crystallin’s fragments isolated from the human lenses help crystallin aggregation in vitro [259,260].

#### 5.2.7. Oxidation

Protein oxidation is a covalent modification of a protein caused either by the direct reaction with reactive oxygen species (ROS) or indirect reaction with secondary by-products of oxidative stress. ROS can lead to oxidation in both amino acid side chains and protein backbone, forming protein–protein cross-links or protein fragments [261]. In the lens, oxidation of methionine (Met) amino acid to methionine sulfoxide or sulfone and cysteine (Cys) to cystine occur [247,262]. The oxidative damage accumulates over time, which occurs at higher rates in the cataractous lens [30,263] compared to normal lenses. The metal-catalyzed oxidation of α-crystallin with H_2_O_2_ caused a significant loss of its chaperone activity [246,264]. Disulfide bonds are sulfur–sulfur bonds that are products of oxidative damage when the thiol (-SH) group of two cysteine amino acids are oxidized, resulting in a net loss of two electrons to the oxidizing agent. Oxidation is an indicator of age-related cataracts in the human lens, because its occurrence highly correlates with the onset of age-related cataracts, especially in the nucleus [30,265,266]. Notably, in young human lenses until approximately 30 years of age, ∼45% of αA-crystallin have an intramolecular disulfide bond [232,265,267,268]. With aging, the amount of disulfide bonds increases up to ∼90% [267], which is a significant constituent of higher molecular weight aggregates [231,235,269], resulting in age-dependent loss of chaperone activity of α-crystallin [20,248].

PTMs decrease the α-crystallin’s chaperone-like activity by the formation of its water-insoluble protein aggregates [230,270]. PTMs of α-crystallin might increase the protein aggregation and insolubilization in the lens [222,224,226]. The water-insoluble α-crystallin in the lens may be the precursor of cataract formation. α-crystallin denatures by modifications, such as PTMs [244,250], and may bind deep into the membranes [79,98]. While PTMs in α-crystallin show their role in cataract formation, the overall role of these PTMs in the α-crystallin binding to the lens membranes leading to the development of cataracts is less clear. However, there are reports that α-crystallin from older [93,96,103] and cataractous lenses [95] that have undergone PTMs did not bind effectively to lens membranes. Such observations warrant more investigations in this area. The high correlation between PTMs in α-crystallin and cataract formation helps to speculate that PTMs likely increase the association of α-crystallin with the lens membrane, promoting cataract progression and development. α-crystallin interacts with membranes likely via hydrophobic interactions, depending on the hydrophobic residues exposed on the surface of α-crystallin oligomer [99,101,108]. It is very likely that different PTMs of α-crystallin alter the structure of α-crystallin differently, changing the proportion of hydrophobic residue on the surface of the α-crystallin oligomer, resulting in a different extent of interactions with the fiber cell plasma membranes. The effect of PTMs of α-crystallin in the association of α-crystallin with fiber cell plasma membrane is an open area of investigation. The detailed research in this area leads to a deeper understanding of cataract progression and development.

## 6. Conclusions and Future Perspectives

α-crystallin has been the “kernel” of small heat-shock proteins in the eye lens. It plays a significant role as a major refractive element and a molecular chaperone in the eye lens. α-crystallin stabilizes other lens proteins against unfolding and misfolding and acts as the first line of defense against lens proteins’ aggregation. Even with these constructive functions, α-crystallin binds to the fiber cell plasma membrane of the eye lens progressively with age and cataract formation.

In this paper, we have reviewed the role of individual lens lipid components, such as major PLs and sphingolipids, Chol, CBDs, integral membrane proteins (MIP26 and connexins), as well as lipid peroxidation and α-crystallin mutations and PTMs in α-crystallin binding to the lens membranes accompanied by light scattering and cataract formation. The binding of α-crystallin to the lipids and Chol/lipid membranes is likely hydrophobic [99,101,108], which occurs between the hydrophobic regions of α-crystallin and the hydrophobic core of the membranes. The lipid headgroup’s size and charge, hydrogen bonding between headgroups, and lipids’ curvature likely modulate the hydrophobic binding [101,108]. Moreover, α-crystallin binding to each lipid and Chol/lipid membrane is saturable, and if compared between different lipid types, the binding is lipid-specific [99,101,108]. The different MMSO and K_a_ of α-crystallin binding to the different lipids (Figure 2) and Chol/lipid (Figure 4) membranes indicate that the change in lipid composition strongly modulates the binding of α-crystallin to the membranes. The increase in Chol content, with the formation of CBDs, within the Chol/lipid membranes inhibits the binding of α-crystallin to the membranes (Figure 6). This indicates that exceedingly high Chol content in the lens membrane plays a positive physiological role in protecting against α-crystallin binding to lens membranes and cataract formation. Additionally, lipid oxidation likely increases the binding of α-crystallin to the lens membranes and possibly promotes cataract formation.

The binding of α-crystallin to the lipids and Chol/lipid membranes changes the physical properties of the membranes [77,78,98], such as mobility parameter and maximum splitting. Increasing α-crystallin concentration decreases the mobility parameter of both the lipids and Chol/lipid membranes [77,78,98], meaning that those membranes become less mobile near the headgroup regions when increasing α-crystallin binds to them. The maximum splitting of a few of the lipid membranes significantly increases with an increase in α-crystallin concentration [101], implying that these membranes become more ordered near the headgroup regions due to the binding of α-crystallin. A crucial integral membrane protein, namely AQP0 or MIP26, binds to the α-crystallin, likely through hydrophobic binding [96], implying a high concentration of MIP26 present in the lens membrane strongly influences the binding of α-crystallin to the lens membranes. Denatured α-crystallin binds deep into the membrane [2,66,79]. α-crystallin binds to other lens proteins forming higher molecular weight complexes (HMWCs), and the association of HMWCs with lens membrane increases with age and cataract [76,77,78]. Moreover, various α-crystallin mutations and PTMs correlate with cataracts [210], implying that these mutations and PTMs likely change the structure of α-crystallin, leading to large-scale crystallin precipitation, followed by association with the lens membranes and cataract formation.

Even with such a plethora of existing knowledge, there still exists some nescience to fundamental questions: How does α-crystallin bind to other proteins in the lens forming HMWCs? How do HMWCs associate with the fiber cell plasma membrane [66], followed by light scattering and cataract formation [59,60,79]? Does α-crystallin have additional regulatory roles? How do α-crystallin mutations and PTMs specifically affect the α-crystallin structure, affecting its binding to the lens membranes? Such fundamental questions warrant detailed studies that help to explain the cause and mechanisms of the molecular-level changes in the lens components that lead to cataract formation and progression. As illustrated in the studies [238,255], MD simulations could provide molecular-level information about the α-crystallin, which could form the basics to understand its association to the fiber cell plasma membrane of the eye lens. However, MD simulations may suffer from the enormous computing resources required for all-atom simulations of the larger molecular complex of α-crystallin and lens membrane. Synergistic approaches of both experiments (e.g., cryo-electron microscopy, NMR spectroscopy, EPR spectroscopy, and cross-linking/mass spectrometry) and simulations are needed, which may provide a molecular-level understanding of α-crystallin association with the lens membrane. A detailed understanding of the mechanism of α-crystallin association with the fiber cell plasma membrane of the eye lens may provide avenues towards the treatment and prevention of cataracts.

## Data Availability

Not applicable.
